# Non-union rates and complications after elective interphalangeal joint arthrodesis of the hand: a cohort study of 366 arthrodeses in 289 patients

**DOI:** 10.2340/17453674.2026.46627

**Published:** 2026-09-14

**Authors:** Karl LINDBERG, Hanna SAND-UHLÉN, Martin CLEMENTSON, Magnus TÄGIL, Deepak Bushan RAINA, Elisabeth BROGREN

**Affiliations:** 1 Department of Hand Surgery, Skåne University Hospital, Malmö; 2 Faculty of Medicine, Lund University, Lund; 3 Department of Orthopaedics, Institute of Clinical Sciences, Lund University, Lund; 4 Faculty of Medicine, Department of Clinical Sciences Lund, Orthopaedics, Lund University, Lund, Sweden

## Abstract

**Background and purpose:**

Interphalangeal joint arthrodesis of the hand is a common procedure, but data on outcomes and complications is limited. We aimed to determine the non-union rate, evaluate risk factors for non-union, and investigate the frequency and distribution of complications across different fixation methods.

**Methods:**

We retrospectively reviewed all interphalangeal joint arthrodeses performed between 2014 and 2022. Union and non-union were defined clinically and/or radiographically. Potential risk factors for non-union were evaluated by logistic regression with cluster-robust standard errors accounting for clustering of multiple arthrodeses within patients.

**Results:**

After exclusions, 366 arthrodeses in 289 patients with ≥ 2 years of follow-up were analyzed. The median age was 63 years (interquartile range [IQR] 57–72) among women and 58 years (IQR 41–69) among men. Non-union occurred in 20 of 366 arthrodeses (5.5%, 95% confidence interval [CI] 3.6–8.3). Men had higher odds of non-union than women, but the difference was not statistically significant (OR 2.43, CI 0.98–6.01); no other characteristic was associated with non-union. Hardware-related complications were frequent, particularly following fixation with K-wires and cerclage, and resulted in hardware removal in 47% of cases. Other complications included superficial infections in 7% of cases, deep infections in 4%, and malunion in 4%.

**Conclusion:**

Elective interphalangeal joint arthrodesis was associated with a non-union rate of 5.5% (CI 3.6–8.3) and few major complications; however, hardware-related problems were frequent and commonly hardware removal was required.

Evidence on arthrodesis of interphalangeal joints (IPJ) of the hand remains relatively limited regarding union rates and associated complications. This may be partly explained by its reputation as a technically straightforward and uncomplicated procedure. However, failed union of an IPJ arthrodesis (non-union) potentially leads to severe symptoms such as pain, instability, and weakness, and often requires revision surgery. Nonunion and reoperation rates vary considerably between studies, ranging from 3% to 20% [1-9].

The traditional fixation methods using K-wires and cerclage are increasingly being replaced by headless compression screws [10-15]. However, as with the introduction of any new surgical technique, it is important to have solid outcome data from the already established methods to enable meaningful comparisons and to justify a change in treatment strategy.

The main aim of our study was to determine the rate of non-unions after elective IPJ arthrodesis. Secondary aims were to evaluate potential risk factors for non-union and to investigate the frequency and distribution of complications across different fixation methods.

## Methods

### Study design

In this retrospective observational cohort study, electronic medical records from Skåne University Hospital between January 1, 2014 and December 31, 2022 were reviewed. Procedures were identified using the Swedish intervention codes NDG46 (arthrodesis of hand or finger joint with internal fixation—interphalangeal joint) but also NDG49 (arthrodesis of hand or finger joint with internal fixation—unspecified joint in hand). If a patient underwent multiple arthrodeses simultaneously, each arthrodesis was counted as an individual case. The minimum follow-up period was 2 years. Because follow-up was determined by the timing of retrospective medical record review rather than scheduled visits, the distribution of total follow-up time beyond this threshold was not systematically recorded. All surgeries were conducted at the Department of Hand Surgery at Skåne University Hospital in Malmö, a specialized center with board-certified hand surgeons and residents in training. Patients were followed at the Department of Hand Surgery with clinical and/or radiographic assessments to determine union or non-union.

The study was reported according to STROBE guidelines.

### Patients

We included patients who underwent distal interphalangeal joint (DIP), proximal interphalangeal joint (PIP), or thumb interphalangeal joint (IP) arthrodesis. Patients younger than 18 years were excluded. Exclusion criteria also included arthrodesis performed due to acute trauma (i.e., as the primary treatment of a recent injury such as a non-reconstructable acute fracture or acute joint destruction), revision surgeries (re-arthrodesis or implant failure converted to arthrodesis), and patients who had received a cortisone injection in the joint less than 3 months prior to surgery. Arthrodesis performed for a post-traumatic condition (e.g., post-traumatic osteoarthritis after a previous, healed injury) was included and categorized under osteoarthritis.

### Variables

Patient records were reviewed by 2 of the authors (HSU and KL). Retrieved data included sex, hand dominance, smoking, diabetes, continuous NSAID use, age at surgery, surgical indication, surgeon’s experience, joint and side, surgical cartilage resection method, surgical fixation method, time to confirmed union, reported complications, and revision surgery. If confirmation of union was not possible because the patient received follow-up at another hospital, failed to attend the scheduled follow-up appointment, or if the available medical records were incomplete or lacked sufficient detail, the case was classified as lost to follow-up. Cases were classified as lost to follow-up only when neither union nor non-union could be established.

### Radiology

Radiographs were examined when available to confirm information on union from the medical records. In cases where only fluoroscopic assessment was performed at the outpatient clinic without saved images, the description of the image documented in the medical records was used.

### Definitions

Union was determined either radiographically, clinically (complete stability and absence of pain), or both. Radiographic union was defined as bridging bone across the arthrodesis site without visible lucency and was determined by a radiologist at the time of imaging; when only fluoroscopic assessment was performed, the clinician’s documented interpretation was used. Confirmed union > 90 days after surgery was defined as delayed union. Non-union was defined as the absence of clinical or radiological signs of union, with no expected progression toward union, as determined by the treating clinician and documented in the medical records. Delayed union and non-union were regarded as separate entities: whereas delayed union was defined by the 90-day threshold already mentioned, non-union was not defined by a fixed time threshold but by the clinical and/or radiographic assessment of arrested healing with no expected progression toward union.

Surgical indication was divided into 4 groups: (i) osteoarthritis (primary and post-traumatic), (ii) inflammatory arthritis (including rheumatoid arthritis, psoriatic arthritis, gout, and undifferentiated arthritis), (iii) contracture (including Dupuytren’s contracture, Boutonnière deformity, and Mallet finger), and (iv) instability (including deep flexor tendon injuries and chronic dislocations). Technique for preparing the joint surfaces was grouped into: (i) gouge nibbling or (ii) electric saw/high-speed burr. Fixation technique was separated into: (i) absolute stability (tension band wiring, intramedullary screw, and plate and screws) or (ii) relative stability (Kirschner wires alone or with cerclage). In tension band wiring, a figure-of-8 cerclage between 2 Kirschner wires converts bending forces into compression; in the relative-stability group, the cerclage was applied without this figure-of-8 configuration and did not provide the same rigid fixation.

Complications were categorized in 5 groups: (i) nonunion, (ii) superficial infection (defined by erythema, swelling, and/or pus in combination with antibiotic prescription), (iii) deep infection/osteomyelitis (clinical infection signs in combination with radiographic or intraoperative evidence of osteomyelitis), (iv) hardware-related complications (pain/irritation or loosening of osteosynthesis material), and (v) malunion (healed arthrodesis in malalignment affecting function and/or appearance). Planned removal of osteosynthesis material, when documented as such in the operative report, was not classified as a complication; only unplanned removal prompted by symptoms (pain, irritation, or loosening) was registered as a hardware-related complication. In ambiguous cases, the event was conservatively classified as not a complication.

### Surgical technique

The joint was exposed through a dorsal approach. Remaining cartilage and sclerotic bone were removed, and the 2 bone ends were fused with compression, ensuring proper alignment and angulation while avoiding gapping. The choice of both joint surface preparation and fixation technique was made by the individual surgeon according to technical preference and experience.

### Sample size and power considerations

As this was a retrospective observational study, a priori sample size calculation was not performed. However, all eligible cases in our department between 2014 and 2022, with a minimum of 2-year follow-up, were included, representing the complete period for which digital medical records were available.

### Statistics

All analyses were performed using STATA, Standard edition, version 18.0 (StataCorp LLC, College Station, TX, USA). Descriptive statistics were used to summarize the characteristics of the cohort and the arthrodeses. Age was not normally distributed and is presented as median and interquartile range (IQR). Differences in age between sexes were assessed using the Mann–Whitney U-test, with effect size expressed as r (Z/√N). Categorical variables are presented as counts and proportions. The non-union rate was determined by calculating the proportion of arthrodeses that did not achieve union among all operated joints. The 95% confidence interval (CI) for proportions was calculated using the Wilson score method. Hand dominance had 43% missing data and was not used in any analysis; other covariates had less than 1% missing data, except joint resection method (7.1%).

Potential risk factors for non-union were assessed by univariable logistic regression, with unadjusted odds ratios (OR) and 95% confidence intervals (CI) estimated for each factor. To account for multiple arthrodeses within the same patient, cluster-robust standard errors at the patient level were used throughout. To assess the potential influence of lost to follow-up (n = 18) on the overall non-union rate, we performed a sensitivity analysis by calculating the non-union rate under two extreme assumptions: that all 18 had united (best case) and that all 18 had been non-unions (worst case).

To investigate the frequency and distribution of complications, postoperative complications were summarized descriptively and stratified by fixation method.

### Ethics, data sharing plan, funding, use of AI, and disclosures

The project was approved by the Swedish Ethical Review Authority (Etikprövningsnämnden, Dnr. 2023-06877-01).

The project was partially funded by a grant from the Kockska Foundation (Greta och Johan Kocks stiftelser, Trelleborg, Sweden).

AI-based assistance (Chat GPT, Anthropic Claude) was used for language editing and for assistance with formatting and implementing tracked revisions in the manuscript. All statistical analyses, interpretation, and scientific content are the authors’ own, and the authors take full responsibility for the final manuscript.

Complete disclosure of interest forms according to ICMJE are available on the article page, doi: 10.2340/17453674.2026.46627

## Results

**Figure 1:**
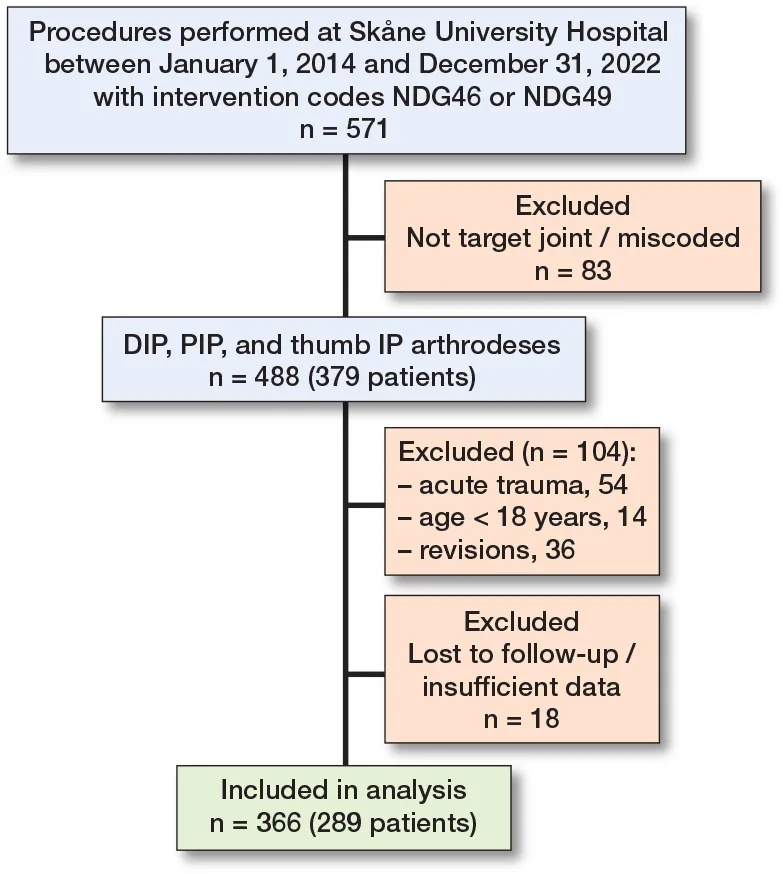
Flowchart illustrating identification, inclusion, exclusions, and final study cohort.

571 procedures were identified. Of these, 83 were excluded at screening as they were not DIP, PIP, or thumb IP arthrodesis (e.g., miscoded cases, wrist arthrodesis), leaving 488 arthrodeses in 379 patients eligible. Of these, 104 arthrodeses were excluded according to predefined criteria, and 18 arthrodeses were lost to follow-up or had insufficient clinical data. This left 366 arthrodeses in 289 patients available for analysis (Figure 1).

The median age at surgery was 63 years (IQR 57–72) among women and 58 years (IQR 41–69) among men (P < 0.001, r = 0.24). Patient characteristics showed a female predominance (66%) with a median age of 63 years (Table 1). Osteoarthritis was the most common indication, followed by inflammatory arthritis, and the DIP joint of the index finger was the most frequently operated on joint. Fixation and surgical characteristics were dominated by K-wire and cerclage fixation (63%) and gouge joint preparation (61%) (Table 2).

**Table 1:** Characteristics of the study population. Values are number (%) of patients (N = 289)

Characteristics	n (%)
Patients	289
Arthrodeses	366
Age at surgery,	
median (IQR)	63 (54–71)
Sex	
Female	190 (66)
Male	99 (34)
Hand dominance	
Right	152 (92)
Left	14 (8.4)
Missing	123
Continuous NSAID use	
No	271 (94)
Yes	17 (5.9)
Missing	1
Smoking	
No	246 (86)
Yes	41 (14)
Missing	2
Diabetes	
No	260 (90)
Yes	28 (9.7)
Missing	1

**Table 2:** Characteristics of the arthrodeses. Values are number (%) of arthrodeses (N = 366)

Characteristics	n (%)
Indication	
Osteoarthritis	207 (57)
Inflammatory arthritis	68 (19
Contracture	75 (21)
Instability	12 (3.3)
Other	3 (0.8)
Missing	1
Digit and joint	
Thumb IP	54 (15)
DIP II	62 (17)
DIP III	58 (16)
DIP IV	27 (7.4)
DIP V	38 (10)
PIP II	24 (6.6)
PIP III	15 (4.1)
PIP IV	30 (8.2)
PIP V	58 (16)
Joint resection method	
Gouge	207 (61)
Saw	133 (39)
Missing	26
Fixation method	
K-wires only	16 (4.4)
K-wires + cerclage	232 (63)
Tension band wiring	82 (22)
Intramedullary screw	29 (7.9)
Plate	7 (1.9)
Surgeon’s experience	
Resident physician	61 (16)
Board-certified specialist	305 (84)

DIP = distal interphalangeal;

K = Kirschner;

PIP = proximal interphalangeal.

Non-union occurred in 20 of 366 arthrodeses, corresponding to a rate of 5.5% (CI 3.6–8.3). In the sensitivity analysis, the non-union rate ranged from 5.2% (assuming no events among missing patients) to 9.9% (assuming all missing patients had non-union), suggesting that the lowest plausible non-union rate was close to the estimated rate (5.2% vs 5.5%) whereas the highest rate was slightly above the estimated CI (9.9% vs 8.3%).

Delayed union (> 90 days) was observed in 30% of cases. Re-arthrodesis was required in 14 cases (3.8%), primarily due to non-union (n = 6, 1.6%) and in the remaining cases due to malunion or other causes (n = 8, 2.2%). Delayed union occurred in 3/29 (10%) after screw fixation, 70/232 (30%) after K-wire and cerclage fixation, 7/16 (44%) after K-wire only, 27/82 (33%) after tension band wiring, and 4/7 (57%) after plate fixation.

The unadjusted non-union rate was higher in men (9.0%) than in women (3.9%), corresponding to an unadjusted OR of 2.43 (CI 0.98–6.01); no other characteristic showed a meaningful association with non-union (Table 3).

**Table 3:** Potential risk factors for non-union expressed as unadjusted OR estimated by univariable logistic regression with cluster-robust standard errors at the patient level. Values are number (%) of arthrodeses (N = 366)

Characteristic	Total, n / n_ref_	Non-union, n (%) / n_ref_ (%)	Unadjusted OR (CI)
Age (per year)	366	–	0.99 (0.96–1.02)
Sex (ref.: female)			
Male vs female	111 / 255	10 (9.0) / 10 (3.9)	2.43 (0.98–6.01)
Continuous NSAID use (ref.: no)			
Yes vs no	18 / 345	1 (5.6) / 18 (5.2)	1.07 (0.13–8.60)
Smoking (ref.: no)			
Yes vs no	53 / 311	3 (5.7) / 17 (5.5)	1.04 (0.28–3.79)
Diabetes (ref.: no)			
Yes vs no	40 / 325	2 (5.0) / 18 (5.5)	0.90 (0.19–4.28)
Surgeon’s experience (ref.: resident)			
Specialist vs resident	305 / 61	17 (5.6) / 3 (4.9)	1.14 (0.33–3.99)
Joint resection method**^a^** (ref.: gouge)			
Saw vs gouge	133 / 207	8 (6.0) / 11 (5.3)	1.14 (0.44–2.93)
Fixation stability (ref.: absolute)			
Relative vs absolute	248 / 118	14 (5.6) / 6 (5.1)	1.12 (0.42–2.96)

CI = 95% confidence interval; OR = odds ratio; Resident = resident physician; Specialist = board-certified specialist.

a Missing data: joint resection method 7.1% (n = 26); all other variables < 1%.

**Figure 2:**
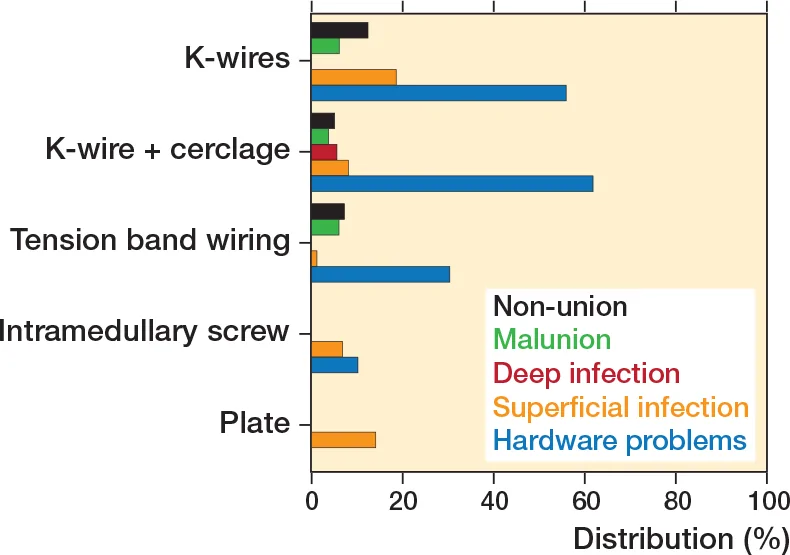
Distribution of postoperative complications stratified by fixation method. Values are percentages of arthrodeses.

Nearly half of all arthrodeses (49%) were associated with symptoms related to osteosynthesis material, making this the most common complication (Figure 2). 47% of arthrodeses underwent extraction of osteosynthesis material, performed either in the operating theatre (n = 77) or in the outpatient clinic (n = 95). Infections were less frequent, with superficial infection in 7.1% and deep infection in 3.6% of cases. Malunion occurred in 4.1%. Overall, elective IPJ arthrodesis was associated with few severe complications, although hardware-related symptoms were frequent.

## Discussion

We aimed to determine the non-union rate, evaluate potential risk factors for non-union, and investigate the frequency and distribution of complications across different fixation methods.

We found a non-union rate of 5.5% following elective IPJ arthrodesis.

Although 18 arthrodeses (4.7%) were lost to follow-up, the sensitivity analysis indicated that our findings were relatively robust to missing data. Men had higher odds of non-union than women in the univariable analysis, but the difference was not statistically significant (OR 2.43, 95% CI 0.98–6.01). No other factor was associated with non-union, including surgeon experience, which has been proposed as a risk factor in earlier studies [2,3]. With only 20 non-union events, the exploratory risk-factor analysis had limited statistical power, and no firm conclusions concerning risk factors can be drawn. Our results are consistent with recently reported figures. Silvano et al. reported a non-union rate of 4.9% in 149 DIP and thumb IP arthrodeses, while Heinonen et al. found a rate of 10% in 310 cases [2,3].

The most notable secondary finding in our study was the high rate of hardware-related problems. Nearly half of all arthrodeses experienced symptoms related to the osteosynthesis material with subsequent need for osteosynthesis removal, particularly following fixation with K-wires and cerclage. This high rate of symptomatic hardware leads to inconvenience for patients requiring follow-up visits and additional healthcare costs.

Approximately 10% of arthrodeses developed infection, mainly among joints fixed with K-wires and cerclage, whereas only occasional superficial infections occurred in the screw group. The relatively high infection rate associated with K-wire-based fixation is consistent with prior reports [16]. Union beyond 90 days occurred in about 30% of cases overall; although most joints ultimately healed, delayed union was more common following K-wire-based techniques and less frequent after screw fixation. Although screw fixation was associated with fewer complications in our cohort, the retrospective design, potential confounding, and the relatively small number of screw fixations limit the ability to draw firm conclusions regarding comparative complication rates.

### Strengths

The main strength of this study was the large and consecutive cohort with all cases having at least 2 years of follow-up.

### Limitations

The main limitation was the retrospective design, with risk of bias by incomplete or inconsistent data. The retrospective design also prevented the use of a standardized time-based definition of non-union. Instead, classification relied on the treating surgeon’s clinical assessment documented in the medical records, which may have introduced some degree of outcome misclassification. Another limitation to our study, and other retrospective studies, is the lack of patient-reported outcome measures (PROMs). While union and complication rates are crucial metrics, factors like pain relief, grip strength, return to work, and overall satisfaction are equally important for surgical decision-making. The absence of these metrics means that the real-life impact of different techniques and outcomes on quality of life remains unclear. Given the single-center design, our findings may not directly generalize to other populations or healthcare systems, although the mix of surgeon experience levels supports applicability to similar high-volume units.

### Conclusion

Elective IPJ arthrodesis appears to yield reliable union and few major complications, but hardware-related symptoms remain frequent.

*In perspective*, the consistently high rate of hardware-related symptoms after Kirschner-wire and cerclage fixation, often necessitating additional procedures, is probably worth considering when selecting a fixation method. Prospective comparative data is needed before any firm recommendation can be made.

## Notes

EB was responsible for both the conceptualization and design of the study. MC, DBR, and MT assisted with the design of the study. Data was collected and sorted by HSU and KL. Statistical analyses were undertaken by KL, HSU, and EB. The manuscript was drafted by KL. Interpretation of the results was done by all authors, who also contributed to critical revision of the manuscript. All authors read and approved the final manuscript.

Handling co-editors: Jeppe Vejlgaard Rasmussen and Philippe Wagner

*Acta* thanks Jarkko Jokihaara and Rasmus Wejnold Troest for help with peer review of this manuscript.
